# The relationship between dietary inflammatory index values and thyroid function in the US adult population: An analysis of the NHANES 2007–2012 cohort

**DOI:** 10.1002/iid3.1016

**Published:** 2023-09-20

**Authors:** Mingzheng Wang, Xiaofeng Lu, Xiaogang Zheng, Junru Liu

**Affiliations:** ^1^ Department of Breast and Thyroid Jinhua Central Hospital Jinhua Zhejiang China; ^2^ Department of Endocrinology and Metabolism Jinhua People's Hospital Jinhua Zhejiang China

**Keywords:** Dietary Inflammatory Index, NHANES, thyroid function

## Abstract

**Objective:**

Researchers have developed the Dietary Inflammatory Index (DII) as a tool to quantify the inflammatory potential of a given diet. Higher DII scores indicated a more proinflammatory diet. While inflammation is known to have a strong impact on thyroid function, the precise nature of the association between DII scores and thyroid function has yet to be clarified. This study was conducted with the goal of exploring this relationship in a representative population of adults from the United States.

**Methods:**

For this study, we used data from the National Health and Nutrition Examination Survey (NHANES). Standardized questionnaires were used to collect demographic and dietary data from the participants, and laboratory tests were used to collect data on the participants' thyroid parameters and other relevant data. Linear regression models and smoothed curve fitting were used to assess the relationship between DII scores and thyroid function, with weighted data analyses and subgroup analyses being conducted as appropriate.

**Results:**

In total, 7712 subjects were recruited from the NHANES 2007–2012 cohort. Their weighted mean age was 44.87 (0.47) years, mean DII score was 1.41 (0.05). Mean FT3 was 3.20 (0.01) pg/mL and mean TT4 was 7.81 (0.03) µg/dL. In adjusted analyses, higher DII values were related to increases in FT3 (*β* = .007; *p* = .027) and TT4 (*β* = .050; *p* = .005) levels. Subgroup analyses showed a negative correlation between FT3 levels and DII scores in a population with high urinary iodine concentrations.

**Conclusion:**

These data indicate that the consumption of a more proinflammatory diet, as evidenced by elevated DII scores, is correlated with significant increases in FT3 and TT4 levels. However, for people with high urinary iodine concentrations, a more proinflammatory diet was associated with lower FT3 levels. Additional research will be vital to clarify the mechanistic basis for these findings.

## Graphical abstract

We explored the relationship between DII and thyroid function using data from the NHANENS database. Our study showed that consumption of a more proinflammatory diet, as evidenced by elevated DII scores, was associated with a significant increase in FT3 and TT4 levels. However, for people with high urinary iodine concentrations, a more proinflammatory diet was associated with lower FT3 levels.

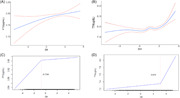

## 1 INTRODUCTION

The Dietary Inflammation Index (DII) is a widely used scoring strategy employed to gauge the potential of overall dietary intake to cause inflammation. This index was initially formulated by Cavicchia et al.^1^ and was more recently updated by Shivappa et al.^2^ Higher DII scores were associated with high levels of several proinflammatory markers such as interleukin‐6 (IL‐6) and C‐reactive protein (CRP), so higher DII scores were considered a proinflammatory diet,^2^, ^3^, ^4^, ^5^, ^6^ in addition to being closely related to the onset and progression of many diseases, including esophageal squamous cell cancer,^7^ squamous cell carcinoma of the head and neck,^8^ cardiovascular disease,^9^ rheumatoid arthritis,^10^ and increased mortality in breast cancer survivors.^11^ Furthermore, the systemic balance of chronic inflammation is more dependent on diet than on drugs, given the frequency of food intake.^12^ Therefore, by reducing the intake of a proinflammatory diet, it may be possible to reduce the extent of some inflammatory symptoms or inflammation‐related disorders.

Thyroid hormone production and signaling activity play a key role in human growth and development, shaping physiological processes such as digestion, respiration, heart rate, and thermoregulation.^13^ The hypothalamic‐pituitary‐thyroid (HPT) axis tightly controls thyroid hormone production and secretion. This central axis initiates thyroid hormone production based on signaling in the paraventricular nucleus of the hypothalamus before signaling from the pituitary and thyroid glands.^14^, ^15^ Adenohypophysis‐synthesized thyroid‐stimulating hormone (TSH) stimulates the biosynthesis and release of thyroid hormones, which include triiodothyronine (T3) and tetraiodothyronine (thyroxine; T4).^16^ Most T3 in circulation is generated via the deiodination of T4 by iodothyronine deiodinases in the periphery.^17^ Thyroglobulin and thyroid peroxidase antibodies (TgAb and TPOAb) have been tied to hypothyroidism and autoimmune thyroid diseases including Hashimoto's thyroiditis.^18^ Thyroid‐infiltrating mononuclear and follicular cell production of inflammatory cytokines has been implicated in Graves’ disease.^19^ IL‐21 and IL‐21 receptor expression levels are elevated in patients with Graves’ disease and Hashimoto's thyroiditis, which are forms of autoimmune thyroid disease.^20^ Patients undergoing hemodialysis and exhibiting inflammatory complications were found in a cross‐sectional analysis to exhibit varying thyroid hormone levels.^21^

These previous studies suggest that inflammation may be related to thyroid function. The specific nature of the link between the DII and thyroid functionality, however, remains to be clarified. Although in recent years, Liu et al. have shown a positive correlation between DII and TT4 levels in the male population, their study did not include a female population and did not exclude participants taking thyroid‐related medications.^22^ Therefore, the objective of this study was to interrogate the link between dietary inflammatory indices and thyroid function in the US adult population using data from the National Health and Nutrition Examination Survey (NHANES).

## 2 METHODS

### 2.1 Study population

The cross‐sectional NHANES collects a wealth of data pertaining to the demographic characteristics, health behaviors, and nutrition of individuals in the United States. Researchers can access NHANES data and related statistics online (www.cdc.gov/nchs/nhanes/). These analyses were performed as per the Declaration of Helsinki.

**Figure 1:**
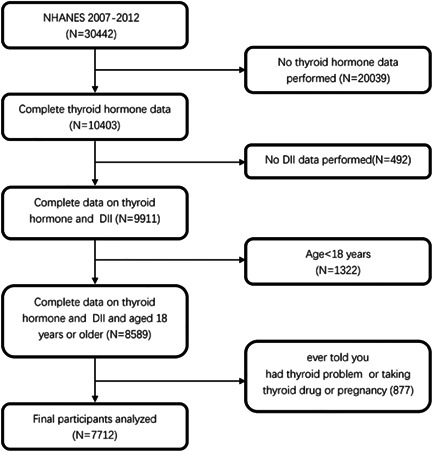
Study flowchart. NHANES, National Health and Nutrition Examination Survey.

This study used 2007–2012 NHANES data approved by the NCHS Research Ethics Review Board (ERB). Written informed consent was provided by all NHANES participants. For these analyses, 7712 subjects aged ≥18 years with complete thyroid function and dietary data used to calculate DII were selected for inclusion. See Figure 1 for details on participant screening.

### 2.2 Measurements

#### 2.2.1 DII

The 24‐h dietary history interview was used to record dietary intake. Dietary intakes were collected and calculated using the USDA dietary survey tool, the Automated Multiple Pass Method (AMPM), which is a fully computerized recall method.^23^, ^24^, ^25^, ^26^ The DII score was calculated from the 24‐h dietary recall data according to the calculation method described in the publication by Shivappa et al.^2^ A greater positive DII score denotes a diet that is more proinflammatory, whereas a lower negative DII score denotes a diet that is anti‐inflammatory. Of the 45 dietary parameters used to compute the DII, 28 were available in the 2007–2012 NHANES cycles for this study, including dietary fiber, β‐carotene, cholesterol, alcohol, protein, carbohydrate, energy, fat, n‐3 fatty acids, n‐6 fatty acids, polyunsaturated fatty acids, saturated fatty acids, and total fat, monounsaturated fatty acids, thiamin, selenium, zinc, magnesium, and iron (Table S1).^22^ Previous research has demonstrated that the DII's predictive power is unaffected by the use of 27 or 28 food characteristics.^27^ In the present study, DII scores were treated as a continuous variable while quartiles were used as a categorical variable to investigate the relationship between DII values and thyroid function.

#### 2.2.2 Thyroid outcomes

The thyroid parameters analyzed included levels of TSH, free triiodothyronine (FT3), free thyroxine (FT4), total triiodothyronine (TT3), total thyroxine (TT4), thyroglobulin (Tg), TgAb, and TPOAb. A third‐generation two‐site sandwich immunoassay with 0.34–5.6 IU/mL reference range was used to measure TSH levels. While the reference range for the two‐step enzyme immunoassay used to measure FT4 levels was 0.6–1.6 ng/dL, the reference range for the competitive binding immunoassay used to measure FT3 levels was 2.5–3.9 pg/mL. TT3 and TT4 levels were also determined using a competitive binding immunoassay with reference ranges of 5.0–12.0 µg/dL and 80–220 ng/dL, respectively. TPOAb and TgAb titers were also analyzed via a sequential two‐step sandwich immunoassay approach with respective reference range values of 0–9.0 IU/mL and 0–4.0 IU/mL. The Access Tg Assay is a single‐step sandwich assay. The NHANES Laboratory/Medical Technologist Procedures Manual provides comprehensive instructions for specimen collection and processing.

#### 2.2.3 Covariates

A standard questionnaire was used to assess participants’ age, race/ethnicity, poverty/income ratio (PIR), smoking history, gender, physical activity, and alcohol history. A mobile examination center (MEC) was used to assess body mass index (BMI). Considering these factors, the importance of iodine for thyroxine synthesis, the fact that iron deficiency impairs thyroid metabolism, and the correlation between thyroid function and renal function, we also included urinary iodine concentration (UIC), serum iron (Fe), and creatinine (Cre) in our study.^28^, ^29^, ^30^, ^31^, ^32^, ^33^, ^34^, ^35^ Non‐Hispanic White, Mexican American, Non‐Hispanic Black, and Other were the four categories used to classify ethnicity. For subgroup analyses, all continuous variables were converted to categorical variables. PIR scores were used to assess socioeconomic levels. We categorized PIR values as <1 (poor), 1–4 (normal), and ≥4 (rich). BMI values were derived by dividing participant weight (kg) by height squared (m^2^). We followed the classification used in a previous study of DII, which classified BMI for individuals older than 18 years as <25 (normal), 25–29.9 (overweight), and ≥30 kg/m^2^ (obese).^36^ To account for participants’ iodine status, which would inevitably alter thyroid function, we classified UIC values as being <100 (iodine‐deficient), 100–299 (normal), and ≥300 g/L (excessive iodine consumption).^37^ Serum iron was categorized as <40 µg/dL, 40–160 µg/dL, and >160 µg/dL, and creatinine was categorized as <0.6 mg/dL, 0.6–1.3 mg/dL, and >1.3 mg/dL, based on the reference ranges of the laboratory tests. Three categories were used to determine marital status: married or cohabiting; widowed, divorced, or separated; or never married. Past 12‐month alcohol consumption was also categorized as never (<12 lifetime drinks), former (≥12 drinks in 1 year but no drinks in the past year or ≥12 lifetime drinks but no drinks in the past year), light (≤2 and ≤1 daily drinks for men and women, respectively), moderate (3 and 2 daily drinks in the past 12 months), and heavy (≥4 and ≥3 daily drinks in the past 12 months for men and women, respectively).^38^ Smoking status categories were never (<100 lifetime cigarettes), former (>100 lifetime cigarettes but not a current smoker), and current. Physical activity was categorized as yes or no, with yes being defined as at least 10 consecutive minutes of walking, bicycling, work, or recreational activity in a typical 1‐week period, and no otherwise. Serum Fe (g/dL), UIC (g/L), and Cre (mg/dL) were the laboratory markers used in this study, all measured by competent technicians using standardized procedures.

### 2.3 Statistical analysis

The Analytical Guidelines (NHANES:2007‐2012) were used to calculate and analyze weighted data. The NHANES data release file contains multiple sample weights, including interview weight (wtint2yr) or examination weight (wtmec2yr), and the most appropriate weight depends on the selected variables of interest. Because the MEC sample is a subset of the interview sample, this analysis was conducted using the combined MEC and examination weight. However, the thyroid examination sample from NHANES 2009–2012 (two survey cycles) is a subset of all participants who were interviewed for the MEC examination. Therefore, the subset weight (WTSA2YR) was used when analyzing data from these two survey cycles. The weight of the survey allows it to be extended to the civilian, noninstitutionalized US population.^39^, ^40^

The relationship between DII and thyroid functionality was described with both unadjusted and adjusted linear regression models. Model 1 was unadjusted for covariates, Model 2 was adjusted for sex, age, and race/ethnicity of participants, and Model 3 was further adjusted for BMI, physical activity, alcohol consumption, smoking status, marital status, UIC, serum Cre, and serum Fe levels. Results were further analyzed using smooth curve fitting. In addition, subgroup analyses and tests for interaction were also performed for each covariate to investigate the independent effect of DII on thyroid function levels. Data are means with standard deviations (*SD*s) or percentages, as appropriate. Effect values are reported as β values with corresponding 95% confidence intervals (CIs). A *p* < .05 served as the cut‐off when defining statistical significance. All analyses were performed with R v4.2.2 using the “nhanesR” package (v0.9.4.2) and the “survey” package (v 4.1‐1).

## 3 RESULTS

### 3.1 Participant characteristics

A total of 7712 participants from the NHANES 2007–2012 dataset were incorporated into this analysis. Participant characteristics stratified according to DII quartiles are presented in Table 1. Of these participants, 4132 (weighted proportion: 53.58%) were male and 3580 (weighted proportion: 46.42%) were female, and the weighted mean (*SD*) age of these subjects was 44.87 (0.47) years. DII scores ranged between −5.28 and 5.47 from least to most proinflammatory, with a weighted mean of 1.41 (0.05), while the weighted interquartile ranges of DII were −5.28 to 0.07, 0.08 to 1.64, 1.65 to 2.94, and 2.95 to 5.47, respectively. Study participants had no clinical thyroid problems and were not pregnant.

**Table 1:** Characteristics of the NHANES (2007–2012) study population in DII quartiles.

	DII					
Characteristics	total	Q1	Q2	Q3	Q4	*p* value
*N*	7712	1643	1858	1977	2234	
DII	−5.28 to 5.47	−5.28 to 0.07	0.08 to 1.64	1.65 to 2.94	2.95 to 5.47	
DII	1.41 (0.05)^a^	−1.20 (0.04)	0.88 (0.01)	2.30 (0.01)	3.67 (0.02)	<.001
TSH (mIU/L)	1.86 (0.03)	1.86 (0.04)	1.85 (0.04)	1.85 (0.05)	1.89 (0.07)	.95
FT3 (pg/mL)	3.20 (0.01)	3.21 (0.02)	3.21 (0.02)	3.21 (0.02)	3.19 (0.01)	.79
FT4 (ng/dL)	0.80 (0.00)	0.79 (0.01)	0.80 (0.01)	0.80 (0.01)	0.80 (0.01)	.38
TT3 (ng/dL)	115.12 (0.65)	114.13 (0.84)	115.07 (0.98)	114.69 (0.87)	116.58 (1.06)	.14
TT4 (µg/dL)	7.81 (0.03)	7.59 (0.05)	7.70 (0.06)	7.86 (0.06)	8.07 (0.06)	<.001
TPOAb (IU/mL)	16.45 (1.54)	13.67 (2.56)	16.72 (2.65)	16.54 (3.40)	18.87 (4.00)	.67
TgAb (IU/mL)	5.58 (0.74)	5.05 (1.34)	5.20 (1.30)	6.18 (1.85)	5.90 (1.02)	.94
Tg (ng/mL)	15.17 (0.48)	14.53 (1.35)	13.92 (0.46)	14.99 (0.75)	17.23 (1.01)	.02
Age (years)	44.87 (0.47)	44.77 (0.91)	44.85 (0.59)	45.43 (0.68)	44.42 (0.55)	.7
BMI (kg/m^2^)	28.40 (0.14)	27.69 (0.27)	28.28 (0.20)	28.82 (0.23)	28.80 (0.16)	<.001
Poverty‐to‐income ratio	2.94 (0.06)	3.23 (0.08)	3.12 (0.08)	2.88 (0.07)	2.55 (0.08)	<.001
UIC (µg/dL)	231.02 (10.92)	200.55 (10.42)	220.25 (11.02)	237.17 (27.43)	266.31 (26.23)	.08
Fe (µg/dL)	88.18 (0.80)	92.11 (1.48)	89.17 (1.23)	87.35 (1.41)	84.09 (1.36)	.003
Cre (mg/dL)	0.88 (0.00)	0.89 (0.01)	0.88 (0.01)	0.88 (0.01)	0.88 (0.01)	.75
Gender						<.001
male	4132 (53.58)^b^	1100 (66.71)	1093 (56.28)	1019 (49.44)	920 (38.84)	
female	3580 (46.42)	543 (33.29)	765 (43.72)	958 (50.56)	1314 (61.16)	
Race/ethnicity						<.001
white	3420 (44.35)	782 (71.46)	830 (68.26)	860 (65.20)	948 (64.52)	
black	1615 (20.94)	278 (8.18)	337 (9.55)	436 (12.73)	564 (15.04)	
mexican	1284 (16.65)	264 (8.01)	358 (10.12)	328 (8.99)	334 (7.21)	
other	1393 (18.06)	319 (12.35)	333 (12.07)	353 (13.09)	388 (13.23)	
Smoke						<.001
never	3874 (53.01)	870 (58.23)	960 (55.65)	975 (53.11)	1069 (50.27)	
former	1803 (24.67)	422 (26.30)	466 (25.25)	467 (24.11)	448 (20.04)	
now	1631 (22.32)	271 (15.48)	344 (19.10)	437 (22.78)	579 (29.69)	
Alcohol user						<.001
never	944 (13.57)	150 (6.29)	191 (8.36)	248 (10.54)	355 (14.21)	
former	1318 (18.94)	213 (11.88)	297 (13.52)	345 (15.81)	463 (19.21)	
mild	2139 (30.74)	566 (39.78)	549 (34.53)	527 (33.08)	497 (26.87)	
moderate	1024 (14.72)	230 (18.69)	262 (19.45)	256 (16.38)	276 (14.86)	
heavy	1533 (22.03)	330 (23.36)	371 (24.15)	412 (24.19)	420 (24.85)	
Marital status						<.001
never married	1328 (18.17)	262 (19.02)	274 (16.85)	364 (19.22)	428 (22.61)	
widowed, divorced, or separated	1582 (21.64)	289 (14.22)	335 (14.93)	406 (17.14)	552 (21.75)	
married, or living with partner	4399 (60.19)	1014 (66.76)	1160 (68.22)	1109 (63.64)	1116 (55.64)	
Physical activity						<.001
no	2011 (26.08)	270 (13.18)	420 (17.51)	572 (23.48)	749 (28.87)	
yes	5701 (73.92)	1373 (86.82)	1438 (82.49)	1405 (76.52)	1485 (71.13)	

Abbreviations: BMI, body mass index; Cre, creatinine; DII, Dietary Inflammatory Index; Fe, serum iron; FT3, free triiodothyronine; FT4, free thyroxine; *SD*, standard deviation; Tg, thyroglobulin; TPOAb, thyroid peroxidase antibody; TSH, thyroid stimulating hormone; TT3, total T3; TT4, total T4; UIC, urinary iodine concentration;

^a^ Continuous variable: weighted mean (*SD*).

^b^ Categorical variables: actual frequencies (weighted percentages).

### 3.2 The relationship between thyroid function and DII

Table 2 shows that DII was positively associated with TT3 and TT4 in the unadjusted model (TT3: *β* = .614, 95% CI: 0.209–1.019, *p* = .004; TT4: *β* = .101, 95% CI: 0.076–0.127, *p* < .001). In model 2, adjusted for sex, age, and race/ethnicity, DII scores were positively associated with FT3 and FT4, similar to the trends observed for TT3 and TT4. However, after further adjustment for other confounders (body mass index, alcohol consumption, smoking, urinary iodine concentration, serum iron, creatinine, and physical activity, poverty/income ratio, marital status), only FT3 and TT4 still remained positively associated with DII (FT3: *β* = .007, 95% CI: 0.001–0.013, *p* = .027; TT4: *β* = .050, 95% CI: 0.016–0.083, *p* = .005), while the same was not true for FT4 and TT3. When DII was used as a categorical variable in quartiles, TT4 levels were significantly higher in the highest quartile compared to the lowest quartile (*β* = .217, 95% CI: 0.032–0.401, *p* = .023).

**Table 2:** The relationship between DII and thyroid function.

	Model 1^a^		Model 2^b^		Model 3^c^	
	*β* (95% CI)	*p* value	*β* (95% CI)	*p* value	*β* (95% CI)	*p* value
**TSH (mIU/L)**						
Total DII	−0.002 (−0.024, 0.019)	.823	0.005 (−0.019, −0.029)	.684	0.012 (−0.016, 0.040)	.394
Categories						
Q1	Reference		Reference		Reference	
Q2	−0.012 (−0.125, 0.102)	.836	−0.002 (−0.116, 0.112)	.974	0.038 (−0.079, 0.155)	.508
Q3	−0.012 (−0.136, 0.112)	.848	0.011 (−0.107, 0.128)	.855	0.076 (−0.072, 0.224)	.302
Q4	0.032 (−0.110, 0.173)	.654	0.074 (−0.087, 0.234)	.360	0.129 (−0.052, 0.311)	.154
*p* for trend		.601		.490		.739
**FT3 (pg/mL)**						
Total DII	−0.001 (−0.007, −0.006)	.822	0.010 (0.004, −0.015)	**.001**	0.007 (0.001, 0.013)	**.027**
Categories						
Q1	Reference		Reference		Reference	
Q2	−0.001 (−0.036, 0.035)	.962	0.017 (−0.016, 0.050)	.309	0.013 (−0.022, 0.047)	.457
Q3	−0.002 (−0.046, 0.042)	.934	0.034 (0.000, 0.068)	**.049**	0.026 (−0.014, 0.066)	.198
Q4	−0.015 (−0.049, 0.018)	.359	0.033 (0.004, 0.062)	**.026**	0.017 (−0.016, 0.050)	.302
*p* for trend		.543		.697		.525
**FT4 (ng/dL)**						
Total DII	0.002 (−0.0002, 0.004)	.074	0.003 (0.0003, 0.005)	**.028**	0.002 (−0.001, 0.005)	.159
Categories						
Q1	Reference		Reference		Reference	
Q2	0.004 (−0.009, 0.018)	.506	0.005 (−0.008, 0.019)	.418	0.003 (−0.011, 0.017)	.681
Q3	0.007 (−0.006, 0.021)	.286	0.009 (−0.006, 0.024)	.227	0.006 (−0.011, 0.023)	.491
Q4	0.010 (−0.002, 0.023)	.104	0.014 (0.001, 0.026)	**.033**	0.009 (−0.006, 0.024)	.212
*p* for trend		.843		.752		.785
**TT3 (ng/dL)**						
Total DII	0.614 (0.209, 1.019)	**.004**	0.735 (0.315, 1.155)	**.001**	0.362 (−0.045, 0.769)	.079
Categories						
Q1	Reference		Reference		Reference	
Q2	0.938 (−1.105, 2.982)	.360	1.074 (−0.926, 3.074)	.285	0.693 (−1.692, 3.079)	.556
Q3	0.556 (−1.458, 2.571)	.581	1.076 (−0.736, 2.888)	.237	0.162 (−2.029, 2.354)	.880
Q4	2.450 (0.298, 4.602)	**.027**	2.825 (0.601, 5.049)	**.014**	0.968 (−1.183, 3.118)	.364
*p* for trend		.176		.198		.611
**TT4 (µg/dL)**						
Total DII	0.101 (0.076, 0.127)	**<.001**	0.078 (0.051, −0.104)	**<.001**	0.050 (0.016, 0.083)	**.005**
Categories						
Q1	Reference		Reference		Reference	
Q2	0.107 (−0.040, 0.255)	.150	0.056 (−0.085, 0.197)	.425	−0.013 (−0.186, 0.160)	.878
Q3	0.270 (0.127, 0.412)	**<.001**	0.186 (0.042, 0.330)	**.012**	0.083 (−0.088, 0.253)	.330
Q4	0.480 (0.329, 0.631)	**<.001**	0.363 (0.209, 0.517)	**<.001**	0.217 (0.032, 0.401)	**.023**
*p* for trend		.051		.09		.153

Abbreviations: CI, confidence interval; DII, Dietary Inflammatory Index; FT3, free triiodothyronine; FT4, free thyroxine; TSH, thyroid‐stimulating hormone; TT3, total T3; TT4, total T4.

^a^ Model 1: no covariates were adjusted.

^b^ Model 2: age, gender, race/ethnicity were adjusted.

^c^ Model 3: age, gender, race/ethnicity, poverty‐to‐income ratio, marital status, body mass index, alcohol use, smoking, urinary iodine concentration, serum iron, creatinine and physical activity were adjusted.

**Figure 2:**
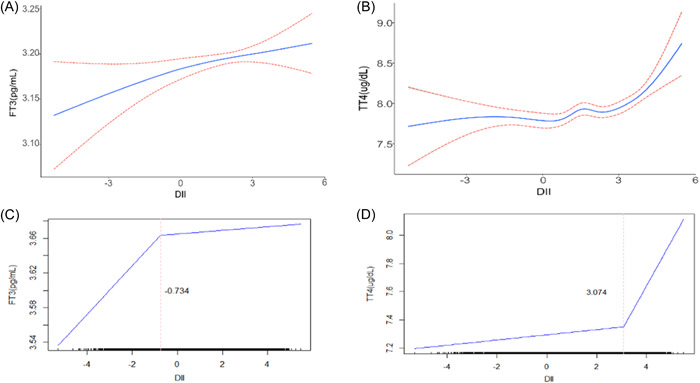
Association between thyroid function and DII in a generalized additive model (GAM) and a segmented linear model. (A) Smoothed curve of FT3 with DII. (B) Smoothed curve of TT4 with DII. The blue solid line represents the smoothed curve fit between the variables, while the 95% CI of this fit is indicated by the pink dashed line. (C) Segmented linear relationship between FT3 and DII. (D) Segmented linear relationship between TT4 and DII. The blue solid line indicates the segmented linear fit between the variables, and the pink dashed line indicates the inflection point K value. CI, confidence interval; DII, Dietary Inflammatory Index.

Further analyses of the generalized additive model revealed that both FT3 and TT4 levels were also positively correlated with DII, and the association between TT4 and DII fit a J‐shaped curve (Figure 2A), whereas a less curved association was evident between FT3 and DII (Figure 2B). The blue solid line represents the smoothed curve fit between the variables, while the 95% CI of this fit is indicated by the pink dashed line. A two‐segment linear model analysis was then applied. As shown in Table 3, the two‐step recursive method showed inflection points at −0.734 and 3.074, respectively (Figure 2C,D). The two‐step and single‐linear models were also compared using the log‐likelihood ratio test.

**Table 3:** Analysis of FT3, TT4, and DII using segmented linear regression.

Models	*β* (95% CI)	*p* value
**FT3**		
**Model I**		
One line effect	0.007 (0.001, 0.013)	.027
**Model II**		
Inflection point (K)	−0.734	
DII < −0.734	0.035 (0.009, 0.061)	.008
DII > −0.734	0.003 (−0.006, 0.012)	.497
*p* for log‐likelihood ratio test		.741
**TT4**		
**Model I**		
One line effect	0.050 (0.016, 0.083)	.005
**Model II**		
Inflection point (K)	3.074	
DII < 3.074	0.018 (−0.008, 0.045)	.125
DII > 3.074	0.290 (0.110, 0.471)	.002
*p* for log‐likelihood ratio test		<.001

*Note*: Model I, one‐line linear regression; Model II, two‐segment regression. β indicates effect size, and 95% CI indicates confidence interval. Adjust for: sex, age, race/ethnicity, marital status, BMI, poverty income ratio, smoking status, alcohol consumption status, UIC, Fe, Cre, physical activity.

Abbreviations: BMI, body mass index; DII, Dietary Inflammatory Index.

### 3.3 Subgroup analyses

**Figure 3:**
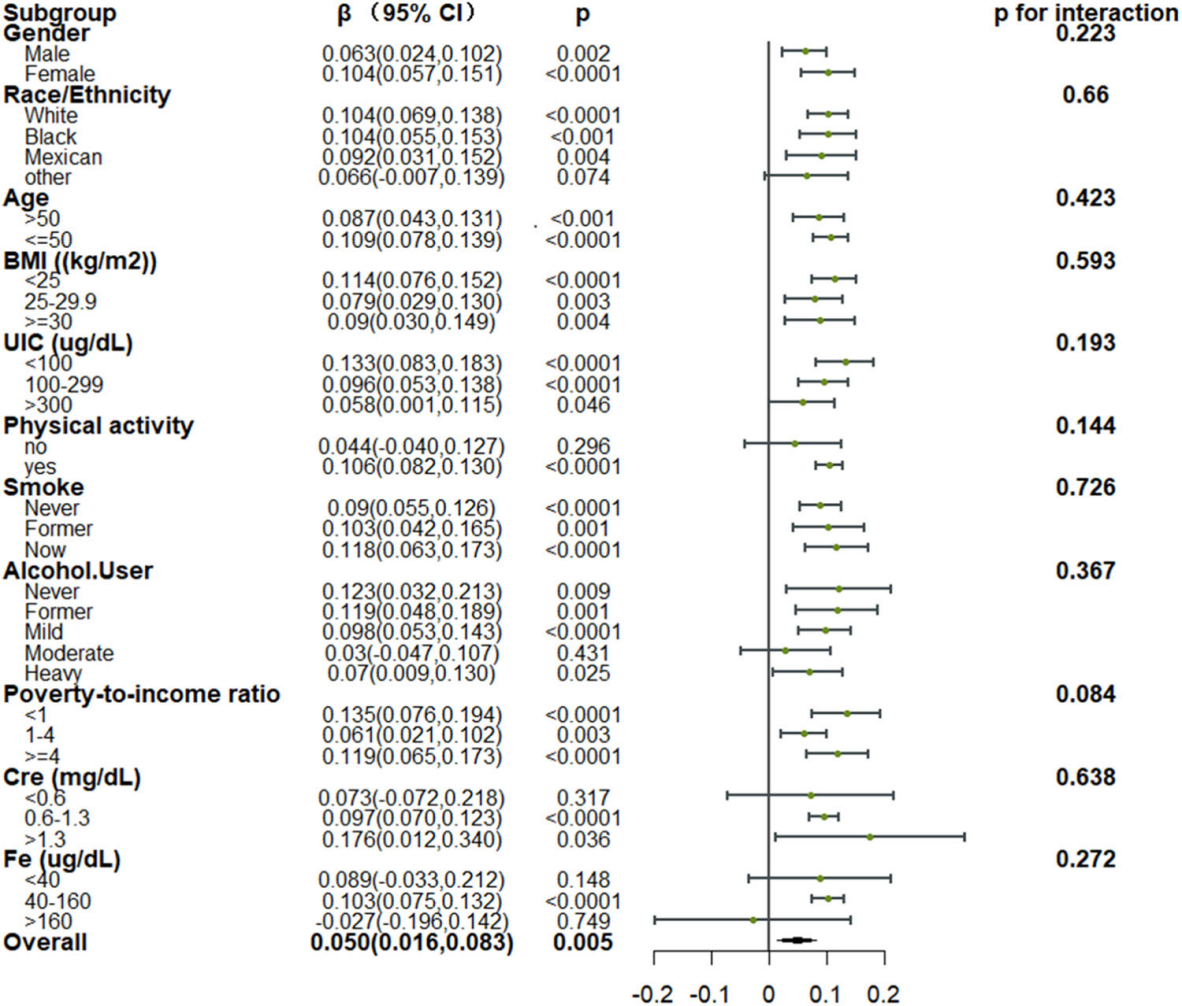
Relationship between TT4 and DII in each subgroup. Each subgroup adjusted for all factors (sex, age, race/ethnicity, marital status, BMI, poverty income ratio, smoking status, alcohol consumption status, UIC, Fe, Cre, physical activity) except the subgroup factor itself. BMI, body mass index; DII, Dietary Inflammatory Index.

**Figure 4:**
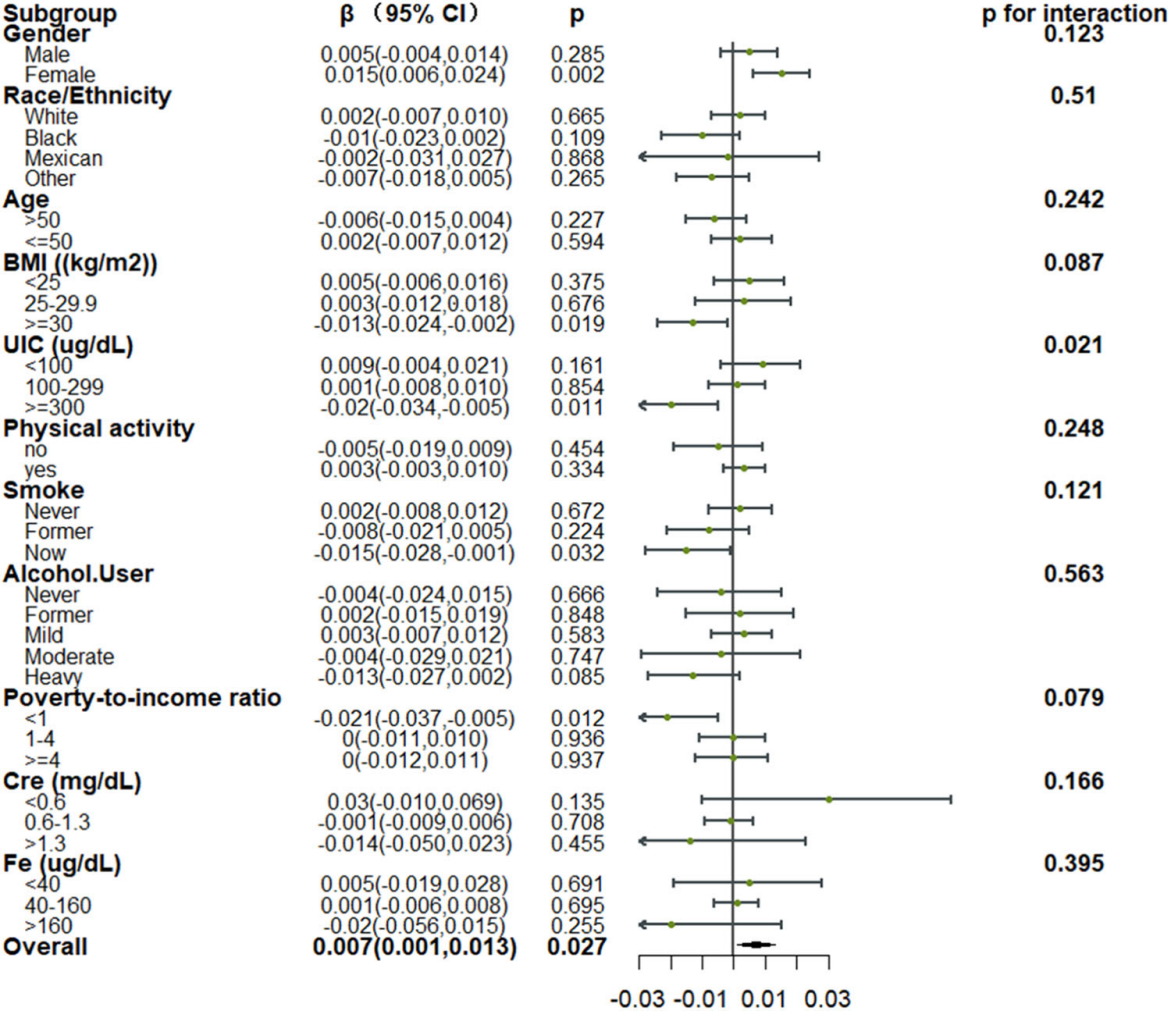
Relationship between FT3 and DII in each subgroup. Each subgroup adjusted for all factors (sex, age, race/ethnicity, marital status, BMI, poverty income ratio, smoking status, alcohol consumption status, UIC, Fe, Cre, physical activity) except the subgroup factor itself. BMI, body mass index; DII, Dietary Inflammatory Index.

Subgroup analyses showed that for TT4 levels, a significant positive association with DII was found in most subgroups, but no significant interaction effect was found (Figure 3). However, for FT3 levels, it is noteworthy that a significant negative association with DII was found in the high urinary iodine concentration subgroup, indicating that lower FT3 levels were associated with a proinflammatory diet, with *p* < .05 for the interaction effect (Figure 4).

## 4 DISCUSSION

This study sought to assess the association between DII and thyroid function by analyzing data from a nationally representative adult cohort from the United States. Individuals with thyroid problems and pregnancy were excluded from our study. These analyses showed that TT3 and TT4 levels were positively associated with DII. This positive association between TT4 levels and DII remained statistically significant after adjustment for relevant confounders, although the same was not true for TT3. We also found that, as with TT4, there was a significant positive association between FT3 levels and DII after adjustment for confounders. When DII was used as a categorical variable in quartiles, TT4 levels in the highest quartile were significantly higher than in the lowest quartile. Overall, proinflammatory diet intake was related to increased TT4 levels and FT3 levels. We also performed further generalized additive and segmented linear model analyses and found a J‐shaped curve relationship between TT4 levels and DII values. The segmented linear model analysis yielded an inflection point K value of 3.074, with a significant increase in TT4 levels with increasing DII after a DII greater than 3.074. In contrast, the curvature of the smoothed curve representing the relationship between FT3 and DII levels was less pronounced and tended to be straight. Although the segmented linear model analysis also yielded an inflection point K value of −0.734, the difference between the two‐segmented linear model and the single linear model was not statistically significant when compared by the log‐likelihood ratio test.

To the best of our knowledge, there have been few attempts to interrogate the relationship between DII values and thyroid function, and our data demonstrate that a proinflammatory diet is related to higher TT4 and FT3 levels. Many factors are known to influence thyroid function, including genetics,^41^ age, gender,^42^ stress,^43^ medication use,^44^ and diet. Iodine intake significantly affects thyroid function, and dietary iodine deficiency is the most frequently identified driver of hypothyroidism.^45^ Several other dietary components can also alter TSH and thyroid hormone levels. Several reports have detected elevated TSH levels after consuming soy isoflavones and soy‐containing foods.^46^, ^47^ The isoflavones in soy can inhibit the thyroid hormone synthesis‐related enzyme TPO, contributing to goiter incidence.^48^ Several studies^49^, ^50^, ^51^ have demonstrated the negative effect of soy isoflavones on thyroid function. Bentvena et al.^52^ found that caffeine blocks the uptake of levothyroxine in hypothyroid patients, thereby affecting thyroid function. Chandra et al.^53^ revealed the potential antithyroid activity of black and green tea extracts. These components are very important factors in DII calculations.

While all of these reports have emphasized the impact of dietary composition on thyroid functionality, these reports have largely centered on specific dietary components rather than the overall inflammatory nature of a given diet. Liu et al. determined that the inflammatory potential of the diet is associated with thyroid function such that in males in the United States, higher DII scores coincided with elevated TT4 levels.^22^ In a gender subgroup analysis of a larger cohort of patients, this study showed that similar to the male population, a proinflammatory diet was associated with elevated TT4 levels in the female subgroup. In the female subgroup, a proinflammatory diet was also associated with higher FT3 levels. However, no significant interaction effects were observed. Subgroup analyses performed based on urinary iodine concentrations indicated that the correlative relationships between DII scores and TT4 levelwere more robust in iodine‐deficient individuals, in line with the report published by Liu et al.^22^ However, no significant interaction effect was found. Notably, in the high urinary iodine concentration subgroup, we found a negative correlation between DII scores and FT3 levels, meaning that a proinflammatory diet was associated with lower FT3 levels in the high urinary iodine population, and the interaction effect was *p* < .05. Physiologically, the main role of iodine is the facilitation of TH biosynthesis within the thyroid.^45^ A lack of sufficient iodine can contribute to hypothyroidism, while excess iodine can result in both hypothyroidism and hyperthyroidism.^54^ Age also correlates with thyroid function, and T4 secretion has been shown to be slightly reduced in the elderly.^55^ Alcohol consumption can adversely affect thyroid cells, although the results of studies focused on the impact of alcohol on thyroid function have yielded varied findings such that alcoholics reportedly exhibit unchanged^56^, ^57^ or elevated TSH levels^58^ and decreased,^58^ unchanged,^59^ or elevated^60^, ^61^ levels of thyroid hormone. Smoking affects thyroid function.^42^, ^62^, ^63^ Iron deficiency impairs thyroid metabolism.^30^, ^31^ Thyroid function correlates with renal function.^33^, ^34^ We also performed subgroup analyses of these factors, such as alcohol consumption, smoking, serum iron, and creatinine, but no significant interaction was found for these factors.

Mechanistically, there is evidence to suggest that varying T3 and T4 levels may be related to the impact of diet on proinflammatory factors including IL‐1, IL‐6, IL‐17, and tumor necrosis factor. Surgery, for example, can provoke a rapid and robust inflammatory response that includes high levels of neutrophil activation and proinflammatory cytokine release, potentially impairing serum thyroid hormone profiles in affected patients.^64^ In a related cross‐sectional analysis performed in New Caledonia, DII was found to be strongly positively associated with the risk of thyroid cancer, with a significant 1.67 odds ratio indicating that compared to patients in the lowest DII tertile, those in the highest tertile face a 67% increase in the risk of thyroid cancer incidence.^65^ Beksac et al.^66^ further confirmed that thyroid cancer development was significantly positively correlated with the levels of CRP and IL‐6. Dietary adjustment that consists of reductions in proinflammatory food intake may thus help protect against thyroid cancer.

There are several limitations to this study. As a cross‐sectional analysis, establishing causal associations between DII and thyroid function was not possible. In the future, longitudinal analyses will be vital to expand on these results. Second, the analyses of thyroid function were performed at a single point in time such that the timing of sample collection may have impacted the levels of thyroid function,^67^ with the lack of any specific collection time potentially hampering the reliable interpretation of these findings. DII calculations were also performed based on 24‐h dietary recall and may thus be susceptible to recall bias and seasonal variability such that they do not accurately reflect true DII values.^68^ Last, other confounding factors not taken into consideration may have influenced these results, such as total energy intake, which is important in determining the overall inflammatory potential of a diet and for which the energy‐adjusted DII was developed.^6^ Even so, these findings offer a robust population‐level analysis of the association between DII and thyroid function among adults in the United States.

## 5 CONCLUSION

These analyses revealed a positive association between the levels of TT4 and FT3 and DII in a nationally representative cohort of adults from the United States. TT4 and FT3 levels tended to be higher in individuals that consumed a more proinflammatory diet and thus exhibited higher DII scores. However, further subgroup analyses showed that higher FT3 levels were associated with lower dietary inflammatory potential in populations with high urinary iodine concentrations. While these results are promising, additional well‐constructed research efforts will be vital to validate these findings and to confirm any causal association between DII and thyroid function.

## AUTHOR CONTRIBUTIONS

**Mingzheng Wang**: Conceptualization; Funding acquisition; Methodology; Project administration; Resources; Supervision; Validation; Visualization; Writing—original draft; Writing—review & editing. **Xiaofeng Lu**: Validation. **Xiaogang Zheng**: Formal analysis. **Junru Liu**: Conceptualization; Funding acquisition; Methodology; Project administration; Visualization; Writing—original draft; Writing—review & editing.

## CONFLICT OF INTEREST STATEMENT

The authors declare no conflicts of interest.

## ETHICS STATEMENT

This study used 2007–2012 NHANES data approved by the NCHS Research Ethics Review Board (ERB). Written informed consent was provided by all NHANES participants.

## Supporting information

Supporting information.

## Data Availability

Researchers can access NHANES data and related statistics online (www.cdc.gov/nchs/nhanes/). The raw data supporting the conclusions of this article will be made available by the authors, without undue reservation.
