# Supplementary material for: The relationship between dietary inflammatory index values and thyroid function in the US adult population: An analysis of the NHANES 2007–2012 cohort
Source: Immun Inflamm Dis. 2023 Sep 20;11(9):e1016. doi: 10.1002/iid3.1016 (PMC10510473; doi:10.1002/iid3.1016)
Supplement: Supplementary file 1 — Supporting information. [file IID3-11-e1016-s001.docx]

**Table 1 Supplementary Table S1 The methodology of dietary inflammatory index calculation**

| **Food parameter** | **Overall inflammatory effect score** |
| --- | --- |
| Energy (kcal) | 0.18 |
| Alcohol (g) | -0.278 |
| Cholesterol (mg) | 0.11 |
| Caffeine (g) | -0.11 |
| Fat (g) | 0.298 |
| Fiber (g) | -0.663 |
| Folic Acid (μg) | -0.19 |
| β-carotene (μg) | -0.584 |
| Iron (mg) | 0.032 |
| Magnesium (mg) | -0.484 |
| Zinc (mg) | -0.313 |
| Selenium (μg) | -0.191 |
| Thiamin (mg) | -0.098 |
| Vitamin A (RE) | -0.401 |
| Vitamin B 6 (mg) | -0.365 |
| Vitamin B 12 (μg) | 0.106 |
| Vitamin C (mg) | -0.424 |
| Vitamin D (μg) | -0.466 |
| Vitamin E (mg) | -0.419 |
| Protein (g) | 0.021 |
| Niacin (mg) | -0.246 |
| Riboflavin (mg) | -0.098 |
| Carbohydrate (g) | 0.097 |
| MUFA (g) | -0.009 |
| PUFA (g) | -0.337 |
| Saturated fat (g) | 0.373 |
| (n -3) Fatty acids | -0.436 |
| (n - 6) Fatty acids | -0.159 |

The Dietary Inflammatory Index (DII) is a tool based on literature and population data to evaluate the potential inflammatory effects of a person's diet using a 24-hour dietary recall. The DII incorporates a comprehensive literature base and standardizes individual intakes to global reference values. It analyzes the impacts of 45 food parameters from 11 different food consumption datasets on six inflammatory markers. In our study, we screened and scored a total of 1943 articles based on these 45 food parameters. Studies showing an increase in pro-inflammatory cytokines (TNF-α, IL-1β, IL-6, and CRP) or a decrease in anti-inflammatory cytokines (IL-4 and IL-10) were scored as "+1", while null scores were designated as "0". These scores were weighted based on study design. We generated Z-scores by comparing reported values to the standard mean and standard deviation. To address the issue of right skewing, we converted the Z-scores to percentile scores. By doubling each percentile score and subtracting "1", we achieved a symmetrical distribution centered around 0 (null) and ranging from -1 (most anti-inflammatory) to +1 (most pro-inflammatory). This centered percentile value, when multiplied by the corresponding "overall inflammatory effect score" for each food parameter, yielded the "food parameter-specific DII score". Finally, we summed all the "food parameter-specific DII scores" to obtain the overall DII score for an individual. Refer to the table below for the "overall inflammatory effect score" of the 28 food parameters included in our study.
